# Six Underutilized Grain Crops for Food and Nutrition in China

**DOI:** 10.3390/plants11192451

**Published:** 2022-09-20

**Authors:** Zongwen Zhang, Jing Zhang, Ping Lu, Bin Wu, Minxuan Liu, Jia Gao, Chunchao Wang, Keyu Bai, Ganggang Guo

**Affiliations:** 1 Institute of Crop Sciences of Chinese Academy of Agricultural Sciences, Beijing 100081, China; 2 Alliance of Bioversity International and International Centre for Tropical Agriculture, Beijing 100081, China; 3 Institute of Agricultural Resources and Regional Planning of Chinese Academy of Agricultural Sciences, Beijing 100081, China

**Keywords:** underutilized crops, grain crops, germplasm, breeding, production, process

## Abstract

Underutilized grain crops are an essential part of the food system that supports humankind. A number of these crops can be found in China, such as barley, buckwheat, broomcorn millet, foxtail millet, oat, and sorghum, which have characteristics such as containing more nutritional elements, being resistant to biotic and abiotic stresses, and having strong adaptability to poor environments. The diversity of these crops provides options for farmers’ livelihoods and healthy food for the population. Although some mentioned crops such as barley, oat, and sorghum are not underutilized crops globally, they could be considered underutilized in China as they were more important in the past and could be revitalized for food and nutrition in the future. This paper reviews current progress in research and development in the areas of germplasm resource conservation, variety improvement, cultivation technologies, processing, and the nutrition and benefits of six underutilized grain crops in China. It is concluded that underutilized grain crops could play a critical role in food and nutritional security in China.

## 1. Introduction

**Figure 1:**
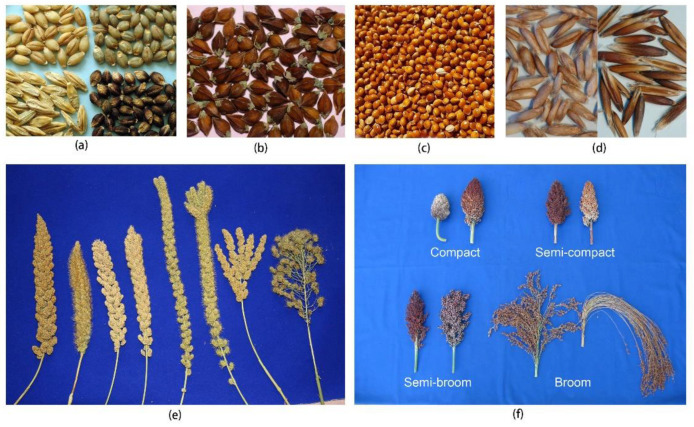
(**a**) Barley grains of different colors; (**b**) buckwheat grains; (**c**) broomcorn millet grains; (**d**) oat hulless/hullness grains; (**e**) foxtail millet earheads; (**f**) sorghum panicles.

**Figure 2:**
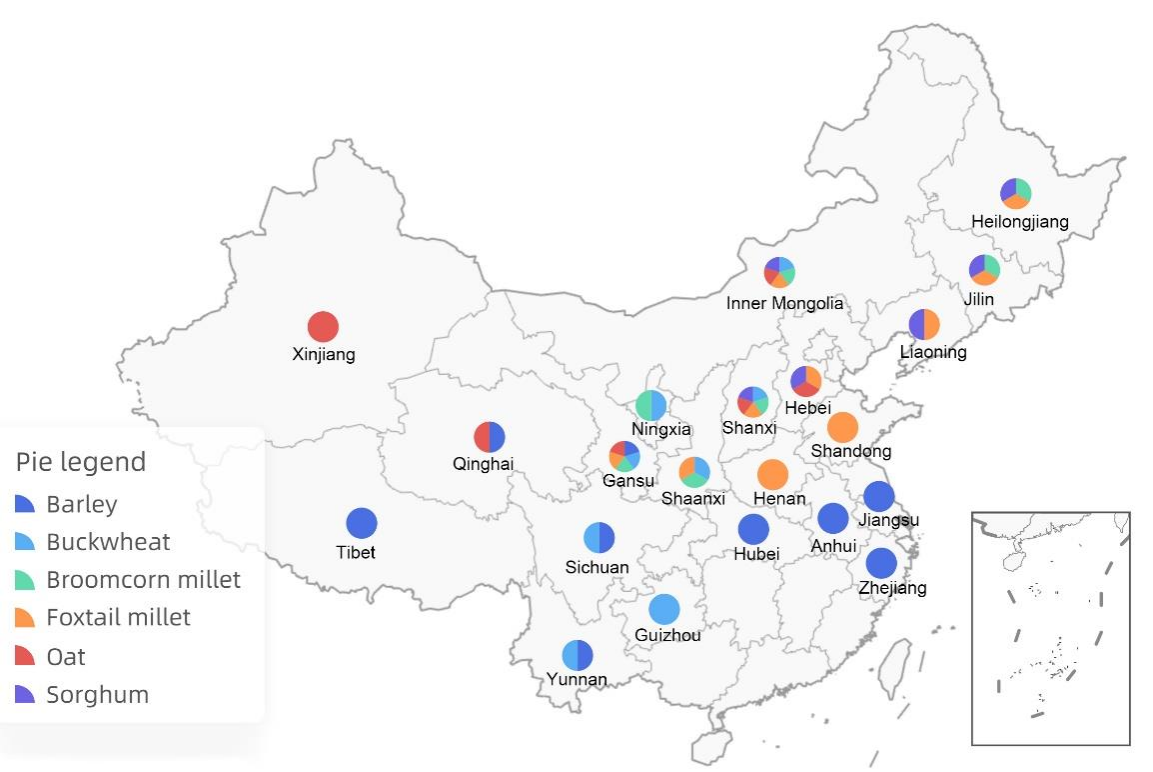
Distribution of six UGCs in different regions of China. Barley: Anhui, Gansu, Hubei, Jiangsu, Qinghai, Sichuan, Tibet, Yunnan, and Zhejiang. Buckwheat: Gansu, Guizhou, Inner Mongolia, Ningxia, Shaanxi, Shanxi, Sichuan, and Yunnan. Broomcorn millet: Gansu, Heilongjiang, Inner Mongolia, Jilin, Ningxia, Shaanxi, and Shanxi. Foxtail millet: Gansu, Hebei, Heilongjiang, Henan, Inner Mongolia, Jilin, Liaoning, Shaanxi, Shandong, and Shanxi. Oat: Hebei, Gansu, Inner Mongolia, Qinghai, Shanxi, and Xinjiang. Sorghum: Hebei, Heilongjiang, Inner Mongolia, Jilin, Liaoning, and Shanxi.

Plants provide most of the food sources for humans around the world. With over 50,000 edible plants, just three of them—rice, maize, and wheat—supply 60% of the world’s food energy intake [1]. China has about 600 species of main cultivated plants [2], of which 43 are used as food crops [3]. Among 117.63 million hectares of food crops grown in China, 96.81 million hectares are occupied by rice (29.92 ha), wheat (23.57 ha), and corn (43.32 ha) [4], and only 20.82 million hectares are used by the other 40 food crops, all of which are considered to be underutilized food crops in China. There are six such crops: barley, buckwheat, broomcorn millet, foxtail millet, oat, and sorghum, all of which are types of cereals or pseudocereals (Figure 1). Grains from these crops are harvested for food and for other purposes; therefore, they are also called underutilized grain crops (UGCs). These six UGCs have a history of cultivation that spans 5000–8000 years and possess diversified landraces that have adapted to various ecological environments, particularly in the arid and cold mountainous areas in the north, northeast, northwest, and southwest regions of China (Figure 2). These crops have very important roles to play:

(1) Adaptability to poor environments: With their short growth period and wide adaptability, these six UGCs can effectively make use of the barren land in high-altitude cold mountainous areas with very low inputs of chemical fertilizers and pesticides, which is critical for managing the vulnerable environments in these areas. This has become an important option to cope with climate change and land degradation.

(2) Source of healthy food: The six UGCs are rich in nutrients, and their protein, vitamin, dietary fiber, and mineral contents are significantly higher than those of staple crops. Some of them are traditional crops that represent sources of both medicine and food. They have become popular health foods in China.

(3) Products of organic agriculture: The varieties of these crops are mostly primary cultivars or landraces, with chemical fertilizers and pesticides rarely being applied during production. They are popularly used in the development of organic agriculture and are highly trusted by consumers.

(4) Source of income for the poor in remote areas: Special products such as folk culture foods made from these six GCs are highly preferred by tourists and consumers from both domestic and international markets. Such products can be developed through local cooperatives or through using a value chain approach and have become an important source of income for local farmers.

(5) Complementary role in food security: The output of these six UGCs accounts for about 3–4% of China’s total grain output. They not only represent an important food source for the local people but also meet the needs of urban residents, enrich food diversity, and play a supplementary role in national food security.

The annual planting areas of the six UGCs reached over 20 million hectares in the 1950s–1960s. Since then, the planting area was reduced to 15 million hectares in the 1980s. Currently, the annual planting area of the six UGCs in China is about 5.5 million hectares, of which about 1.2–1.5 million hectares are taken up by foxtail millet and 0.6–1.0 million hectares are taken up by each of the other UGCs. The total annual output is about 7–8 million tons. The extension of high-yielding crops such as rice, wheat, and corn promoted by the policies on securing national food security is the main reason causing the dramatic drop in the growing area of the six UGCs in China. However, efforts have been made to promote the six UGCs, and considerable progress in research and development has been achieved in the areas of germplasm resources, breeding, production, and processing.

The market demand for the six UGCs in China has greatly increased in recent years. However, the potential of UGCs for food and nutrition has yet to be well realized, and the gaps existing in various areas should be filled through strengthened efforts in research and development. UGCs will play a more important role in enriching dietary diversity and in improving food and nutrition security in the future.

## 2. Profiles of Underutilized Grain Crops

### 2.1. Barley

Barley (*Hordeum vulagre* L.) was domesticated in the Fertile Crescent in about 8000 B.C. [5]. The remains of barley grains from different archaeological sites in China show that barley was domesticated about 5000 years ago, indicating that the Qinghai–Tibet Plateau of China is one of the centers for domestication of hulless barley (qingke) [6,7]. There is a two-rowed subspecies (*H. vulgare* susbp. *distichum* L.) and a six-rowed subspecies (*H. vulgare* subsp. *hexastichum* L.). Barley is widely cultivated in China. In the northern part of the country, barley is sowed in spring as a single-season crop. In the Qinghai–Tibet Plateau, barley is grown on land above altitudes of 3000 m, with an obvious temperature difference between day and night and a short frost-free period, and the multi-rowed hulless barley (qingke) is dominant. In the south of the Yellow River and Huaihe River, barley is planted in autumn as a secondary crop [8]. The cultivated area of barley in China reached 6.5 million hectares in the 1970s. Currently, it remains at about 1 million hectares annually. The yield of barley has increased from 1522.5 kg/ha in the 1970s to 3990 kg/ha at present. Barley is a multi-purpose crop. Its grains are used for food, winemaking, and feed. Hulless barley (qingke) is a staple food for the Tibetan people. In other parts of the country, barley is usually cooked for porridge mixed with rice. China has a long history of making alcoholic drinks with barley, such as wine and beer. Specifically, barley contains more β-glucan (>5%), a dietary fiber that can help to promote human health.

### 2.2. Buckwheat

Buckwheat (*Fagopyrum* spp.) is a pseudocereal that originated in the southwest of China [8,9]. There are two cultivated species, namely sweet buckwheat (*F. esculentum* Moench.) and Tartary buckwheat (*F. tataricum* (L.) Gaertn.). More than 20 wild species have been found in southwestern China [10]. Buckwheat is characterized by tolerance to poor soil and cool weather conditions. Its growth period is short, generally 60–90 days, at which point it reaches maturity. Buckwheat can be grown throughout the country. However, sweet buckwheat is mainly grown in northern China, including in Inner Mongolia, Gansu, Shanxi, Shaanxi, and Ningxia, while bitter buckwheat is mainly grown in southwestern China, including in Guizhou, Sichuan, Yunnan, Chongqing, and Hunan [8]. The annual planting area is 0.6–0.8 million hectares, and the total output is 0.5–0.6 million tons [11]. The yield of buckwheat is low, only about one ton per hectare. Buckwheat grain is rich in protein, vitamins, minerals, plant cellulose, and other nutrients, and specifically, bitter buckwheat contains flavonoids that are mainly composed of rutin, which has pharmacological effects for reducing blood fat and cholesterol and for preventing cardiovascular diseases [12]. Therefore, buckwheat is used for multiple purposes. The flour from the seed can be used to make a variety of foods, and the straw and leaves can be used as fodder and raw medical materials.

### 2.3. Broomcorn Millet

Broomcorn millet (*Panicum miliaceum* L.) originated in China. There is one cultivated species and one semi-wild-type species widely distributed throughout the country. The remains of broomcorn millet in ancient sites in Shaanxi, Shanxi, Gansu, Inner Mongolia, and other places can be traced back to about 8000 years ago, indicating that it is one of the earliest crops domesticated in China [13]. Broomcorn millet is mainly characterized by its early maturity and tolerance to drought and poor soil. It is mainly distributed in the northeast, northwest, southwest, and other arid areas in the northern part of China. In the 1950s, the sown area was 2 million hectares nationwide; since then, it has gradually decreased [8]. At present, the sown area in China is about 1 million hectares. Its yield ranges from 2250 kg/ha to 3000 kg/ha. Broomcorn millet is used for food as well as for winemaking. Its stalks and leaves can be used as forage for animals. Broomcorn millet can be made into a variety of snacks of different flavors. In the north, it is popular to use broomcorn millet to make a cake for Chinese New Year that is deeply loved by local people.

### 2.4. Foxtail Millet

Foxtail millet (*Setaria italica* (L.) P. Beauv.) originated in China in the basin of the Yellow River and has a cultivation history of more than 7300 years [7]. Foxtail millet is mainly distributed in the north of the Yellow River and Huaihe River, including in the provinces/autonomous regions of Henan, Hebei, Shandong, Inner Mongolia, Northeast, Shaanxi, and Gansu. Foxtail millet is a typical environmentally friendly crop that is grown using the dry farming system in mountain areas. In the 1950s, the cultivated area of foxtail millet in China was as high as 15 million hectares, but later, it was gradually replaced by wheat, rice, and corn. At present, the annual planting area is about 1.2 million hectares [14]. The provinces or autonomous regions with the largest planting area are Hebei, Shanxi, Inner Mongolia, Shaanxi, Liaoning, Henan, Shandong, Heilongjiang, Gansu, and Jilin. The yield ranges from 4500 to 7500 kg/ha. Foxtail millet has the biological characteristics of resistance to drought and tolerance to poor environments [15]. It is rich in nutrients such as protein, fat, amino acids, and minerals that are present in much higher concentrations than in staple crops [8]. Millet is easily digested and absorbed by the human body no matter how it is cooked, so it is a good nourishing food for pregnant women, children, the elderly, and the weak. Foxtail millet straw is a high-quality forage for cattle due to its good palatability, rich nutrition, and easy digestion and absorption.

### 2.5. Oat

There are two hexaploid species of oat (*Avena* spp.) that are cultivated, namely *A. sativa* L., which originated in the Middle East, and *A. nuda* L., which originated in China [16]. One wild species *(A. fatua* L.) is widely distributed in China. Oat has been cultivated in China for more than 2000 years. It is mostly grown in northern regions, particularly in Inner Mongolia, Hebei, Shanxi, Gansu, Ningxia, and Xinjiang. In general, hulless oat is characterized by multiple florets compared to the single floret on hullness oat. Both hulless and hullness oats are strongly tolerant to poor soils. They are critical for the livelihoods of farmers in the dry areas in the northern and northwestern parts of the country. Annually, oat was grown on about 1.5 million hectares during the 1950s and 1960s. In the 1970s, the oat cultivation area decreased due to the adoption of high-yielding rice, wheat, and corn. Currently, the annual cultivation area is about 0.67 million hectares, with a total annual production of 0.6–0.8 million tons. The protein and fat contents in oat grain are about 15.0% and 8.5%, respectively. The contents of phosphorus, iron, and calcium in grain are higher than those of any other cereal crop. Specifically, oat contains more β-glucan and is considered as a healthy food for people suffering from high blood pressure, high blood fat levels, and diabetes.

### 2.6. Sorghum

Sorghum (*S. bicolor* L.) originated in Africa and was introduced to China through India about 10–15 centuries ago. It was first planted by ethnic minorities in southwestern China and was later popularized throughout the country. Sorghum has the characteristics of high light-use efficiency, strong stress resistance, and high yield. It is widely cultivated in the southern part of China but is mostly planted in scattered areas. It is mainly grown in the temperate regions of western and northeastern China, including in Jilin, Liaoning, Heilongjiang, Inner Mongolia, Hebei, Shanxi, Shaanxi, Ningxia, and other arid areas. In 1952, the cultivation area was about 9.39 million hectares, which was the historic maximum planting area for sorghum in China. In 2018, the national planting area was about 0.72 million hectares, with a total production of 3.45 million tons [17]. Before the 1980s, sorghum was mainly used for food by processing sorghum rice or flour to make noodles, rolls, pancakes, steamed cakes, and sticky cakes. Since then, sorghum has mainly been used as feed and as raw materials to make wine and to extract starch and sugar.

## 3. Germplasm Resources 

### 3.1. Conservation 

#### 3.1.1. Acquisition of Germplasm

UGC germplasm resources have been collected throughout the country since the 1950s, starting when China conducted the first national collecting program for crop germplasm resources with support from the Ministry of Agriculture. UGC landraces were mainly collected from producing areas in the relevant provinces and regions. In the 1980s, the second national collecting program was implemented, and 60,000–70,000 samples of landraces and breeding varieties and advanced UGC lines were collected from rural communities and from research organizations. At the same time, the National Crop Genebank was constructed for the ex situ conservation of crop germplasm resources in the country [18]. The third national collecting program has been going on since 2015, and more remote areas in the country are being explored in more depth for the collection of not only landraces but also wild populations, particularly those for barley, buckwheat, and millet. China has also introduced some UGC germplasms from foreign countries, such as barley accessions from ICARDA, Canada, and France; oat from Canada, Germany, and Russia; and buckwheat from Nepal. The collected UGC samples were identified, and the catalogs of UGC germplasm resources were compiled with passport information and partial agronomic trait data.

#### 3.1.2. Ex Situ Conservation 

All accessions listed in the UGC germplasm resource catalogs were multiplied by the relevant organizations for ex situ conservation under the coordination of the Institute of Crop Sciences of the Chinese Academy of Agricultural Sciences (ICS-CAAS) with support from a national program for the ex situ conservation of crop germplasm resources. Methodologies for multiplying the accessions of crops, particularly those of open-pollinated crops such as sweet buckwheat, were developed by collaborative research between the ICS-CAAS and the International Plant Genetic Resources Institute (IPGRI) [19], followed by the development of guidelines for the multiplication and regeneration of each UGC [20]. Seed samples of UGC accessions were dried to a moisture content of 6% and were sealed in aluminum containers and stored in the National Crop Genebank at −18 °C for long-term conservation and at −10 °C for characterization and distribution purposes [21]. Up until now, more than 85,800 UGC accessions have been conserved in the National Crop Genebank (Table 1). The largest collection is foxtail millet, followed by barley and sorghum. Among the total collections, landraces account for over 80%.

#### 3.1.3. On-Farm Management 

At present, the on-farm management of crop germplasm resources is still in the preliminary stages in China. The concept of on-farm management was first tested in China by the IPGRI in collaboration with the Chinese Academy of Sciences (CAS) for the management of buckwheat diversity in communities in Liangshan, Sichuan province. It was found that by improving the economic value of the local varieties of bitter buckwheat through linking farmers to markets, the diversity of bitter buckwheat can be continuously maintained and improved by farmers [22]. A national non-governmental organization called the Farmers’ Seed Network has initiated the on-farm conservation of foxtail millet in the traditional dryland farming systems in Aohan, Inner Mongolia. Working with farmers, enterprises, and the local government, more than a hundred traditional varieties of foxtail millet were conserved and protected through ecological farming approaches and by the Global Important Agricultural Heritage System as well as through the establishment of the community genebank [23]. The FAO and the Ministry of Agriculture and Rural Affairs are implementing a GEF project for the Participatory On-Farm Conservation and Sustainable Use of Gene Diversity of Native Crops in China, including millet and oat, which aims to promote the relevant policies and actions for the on-farm management of native crops. With the extension of the six UGCs in cultivation, there will be more opportunities for farmers to use local varieties that are adaptable to different environments. Good practices such as diversified farming systems and low or even no chemical fertilizer or pesticide input during the production of these crops will promote the on-farm management of their genetic diversity. At the same time, the national crop genebank could become involved by repatriating some UGC landraces to the communities from which they were originally collected and could allow them to continue the evolutionary process of natural and artificial selection, greatly contributing to the conservation and sustainable use of diverse UGCs.

### 3.2. Characterization 

#### 3.2.1. Descriptors and Data Standards

To characterize UGC collections, relevant Chinese experts developed descriptors and data standards for barley [24], buckwheat [25], broomcorn millet [26], foxtail millet [27], oat [28], and sorghum [29] that provide the principles and guidelines for evaluating agronomic traits of UGC accessions. The basic principles of characterization should be able to promote access to and the sharing of germplasm resources and should be able to unify the standards for measuring and scaling specific traits of accessions in the field and lab. Over 100 descriptors for each UGC were defined and classified into passport information, plant traits, quality traits, abiotic resistant traits, biotic resistant traits, etc. Common passport descriptors were defined for all crops, including accession number, genebank number, collecting number, accession name, family, genus, species, country of origin, province of origin, sample source, donor institute, donor accession number, pedigree, breeding institute, releasing year, breeding methods, altitude, and longitude and latitude. Characterization of UGC accessions was focused on plant traits, quality traits, abiotic resistant traits, and biotic resistant traits.

#### 3.2.2. Plant Traits 

Plant traits were evaluated for all of the UGC cereals, including seedling leaf color, plant height, stem diameter, stem color, leave length and width, leaf number, flag leaf length and width, tiller number, nodes of stem, ear/panicle length and width, ear/panicle shape, awnedness of spikes, seed weight per ear/panicle, seed coat color, and 1000-grain weight. The following unique plant traits of buckwheat were evaluated: plant branch number, leaf color, leaf blade shape, inflorescence compactness, number of flower clusters per cyme, number of cymes per plant, and flower color. All of these plant traits were measured and recorded for the UGC accessions stored in the National Crop Genebank. The analysis showed that the considerable variation among the accessions of each crop was presented in different characters; for example, the analysis on barley accessions showed that plant height ranged from 52 cm to 119 cm, grain number ranged from 17 to 35 for two-rowed accessions and from 47 to 90 for six-rowed accessions [30], and plant height ranged from 99 cm to 205 cm for sweet buckwheat accessions and from 36 cm to 200 cm for bitter buckwheat [11]. Wang et al. [31] evaluated 15 plant traits of 878 accessions of foxtail millet and found that the level of phenotypic diversity of the Chinese accessions was higher than that of the introduced accessions, especially in terms of the grain weight per main stem, panicle length, panicle diameter, plant height, stem node number, and growth period, and lower in cultivars than in traditional varieties.

#### 3.2.3. Quality Traits

Quality traits are traits related to nutritional elements, including the protein, fat, carbohydrate, vitamin, mineral, and dietary fiber contents in the grains of UGCs. Using standard methods, about one-third of accessions of the UGCs were evaluated for the common quality traits, including proteins, fats, and carbohydrates. To further understand the nature of these quality traits, some important amino acids such as lysine are usually analyzed to determine the protein composition, unsaturated fatty acids such as linoleic acid were determined for the fat composition, and soluble and insoluble sugars were determined for the carbohydrate composition. A small proportion of UGC accessions was analyzed for the vitamin, mineral, and/or dietary fiber contents. For example, vitamins A and E were analyzed in buckwheat and foxtail millet, and calcium, potassium, sodium, iron, zinc, and other vitamins and minerals were analyzed in some accessions of UGCs. Generally, the protein, mineral, and dietary fiber contents in UGCs are higher than in staple crops. More information on the quality traits will be further presented in Section 6.

#### 3.2.4. Resistant Traits

About 20–30% of the UGC accessions were evaluated for traits making them resistant to pests and diseases and/or tolerant to biotic and abiotic stresses. Different resistance traits were evaluated for different UGCs, such as resistance to smut and rust in all cereals and resistance to strip and scab in buckwheat. Specifically, some barley accessions were evaluated for stripe, scab, yellow mosaic virus, yellow dwarf virus, and aphis; buckwheat was evaluated for powdery mildew, downy mildew, brown spot, and blight; foxtail millet was evaluated for downy mildew and blast; and sorghum was evaluated for leaf blight and corn borer. Considerably resistant UGC accessions were identified in different crops, such as accessions resistant to yellow dwarf viruses in barley [32], resistant to ring rot in buckwheat [33], resistant to blast in foxtail millet [34], tolerant to salt in barley and oat [35,36], and tolerant to drought in foxtail millet [37].

### 3.3. Genetic Identification

#### 3.3.1. Methods and Tools

Molecular markers are widely used in genetic identification and plant genome analysis. There are many types of molecular markers, examples of which include restriction fragment length polymorphisms (RFLPs), amplified fragment length polymorphisms (AFLPs), simple sequence repeats (SSRs), Diversity Array Technology (DArT) markers, and single-nucleotide polymorphisms (SNPs), which are useful tools for exploring genetic variation, determining the relationships between populations, and identifying genes in the genetic materials of UGCs. Genome sequencing is increasingly used in the conservation and identification of crop germplasm resources [38]. Whole genome sequencing has become a powerful tool to identify RNA and DNA variations in the plant genome [39,40]. Chinese scientists use these tools to reveal genetic diversity and to identify genes in a wide range of agronomically important crop species, including UGCs.

#### 3.3.2. Genetic Diversity Assessment

Using AFLP markers, Qi et al. [41] found that 74 oat accessions present high genetic diversity according to rich AFLP polymorphism, which could differentiate the geographical origins of different oat accessions. Using ISSR markers, Zhao et al. [42] found that the highest genetic variation was presented by the landraces of bitter buckwheat from Yunnan, followed by those from Guizhou and Hubei. By using SSR markers, Shi et al. [43] found a high level of genetic diversity in buckwheat and its wild species and determined their genetic relationships; Zhang and Zhang [16] differentiated barley cultivars from landraces and separated the domestic accessions from the introduced accessions; Qing et al. [44] analyzed the genetic diversity of foxtail millet varieties released from the 1980s to the 2010s in the summer sowing region of northern China and indicated a constitutive drop in the genetic variation in the cultivars over the last few decades. Liu et al. [45] revealed levels of diversity in the accessions of broomcorn millet and found a close correlation between the geographical regions and genetic diversity of accessions, and Li et al. [46] found a high level of genetic differentiation among different geographical sorghum populations.

#### 3.3.3. Gene Mining

Quantitative trait loci (QTLs) for the important agronomic characteristics of UGCs were identified using genetic linkage maps developed using different molecular markers. With the genetic linkage maps developed on the SSR markers, 17 QTLs associated with grain width, grain length, and 1000-grain weight were identified in oat [47], and two main-effect QTLs related to plant height were observed in foxtail millet [48]. Genome-wide association studies (GWAS) have been used to identify the association between SSR markers with the nudity of oat grain [49] as well as with the photoperiod sensitivity of foxtail millet cultivars [50] and the relationships between DArT markers and the response of Tibetan wild barley genotypes to drought stress [51]. In recent years, whole genome sequencing has been used to mine useful genes in UGCs. Transcriptome sequencing analysis suggests that the gene *FLS1* (flavonol synthase 1) is associated with rutin accumulation in filling-stage seeds of buckwheat species [52] and in several genes in response to salinity stress in oat [53]. By comparing the buckwheat genomes of 34 wild golden buckwheat accessions across two morphologically distinct ecotypes, the candidate genes *FdMYB44* and *FdCRF4*, which are putatively involved in flavonoid accumulation and in the differentiation of plant architecture, were identified [54]. The application of BSA-Seq based on the construction of reduced-representation libraries and allele frequency analysis permitted the barley pale-green (*pg*) gene to be anchored on chromosome 3HL [55].

### 3.4. Access and Distribution 

With the support of the Ministry of Agriculture and Rural Affairs, the Chinese Academy of Agricultural Sciences has coordinated with relevant research organizations to multiply six UGC germplasm resources stored in the National Crop Genebank for access, distribution, and utilization. To access UGC germplasm resources, users may inquire about the germplasm resources of interest on the website of the Chinese Crop Germplasm Resources Information System (www.cgris.net, accessed on 18 September 2022) and send the request directly to the National Crop Genebank (address: No. 12, Zhongguancun South Street, Beijing 10081, China). The six UGC germplasm resources can also be shared and utilized through joint evaluation and cooperative research. The National Crop Genebank provides 3000–5000 accessions of UGC germplasm resources to relevant organizations and individuals every year, and these are mainly used for the characterization and screening of elite materials, variety improvement, and basic research. In recent years, the genebank frequently organized exhibition and exchange fairs for elite UGC germplasm resource accessions with high yield, high quality, strong disease resistance, and good quality to be viewed and chosen by breeders and other researchers.

## 4. Breeding

### 4.1. Breeding Objectives

Breeding objectives not only involve the selection of parental materials but also affect the techniques for selecting and testing the new varieties. Therefore, a clear goal and direction must be set out in advance for all UGC breeding programs. At present, the most important breeding objectives for new varieties of UGCs are high yield, high quality, stress resistance, disease resistance, and suitability for mechanization and processing [56,57]. However, different crops have different characteristic requirements for the adaptation to targeted production areas and for meeting the demands of special markets, such as good brewing quality for barley and sorghum, a high β-glucan content for oat, and earlier maturity for all crops in mountainous areas.

#### 4.1.1. High Yield

High yield is a comprehensive expression of the growth and development of a variety under specific conditions. It is mainly determined not only by genetic factors but also by environmental factors. Yield potential is closely associated with many plant traits, particularly seed weight per unit, seed number per ear/panicle, and number of ears/panicles per unit area. The rate of milled grain is another important yield indicator, which is the true high yield when both the grain yield and the rate of milled grain are high.

#### 4.1.2. Resilience

The resilience of varieties will be affected by those traits that can maintain minimum changes between environmental conditions, including climatic conditions and cultivation inputs, which have a great influence on the stability of the yield of varieties. Therefore, new varieties should have strong stress resistance and adaptability, mainly for drought, cold, lodging, diseases and pests, and poor soil.

#### 4.1.3. High Quality

High quality mainly refers to the ability to maintain the original quality components of UGCs, such as high protein, vitamin, and micronutrient contents. In addition, the prominent feature of UGCs is that they contain functional ingredients, such as rutin in buckwheat and β-glucan in barley and oat. High-quality varieties are particularly preferred by processing companies for use as raw materials to produce healthy products.

### 4.2. Breeding Methods

#### 4.2.1. Systematic Selection

Systematic selection is popularly used to select natural mutants from existing varieties and germplasm resources according to breeding objectives. This method involves pure-line selection and mass selection. Chinese scientists usually use this method to improve the UGC landraces by selecting superior individual plants from large populations and to form new populations with improved traits [58]. It also involves the selection of super lines from the offspring of a cross between two genetically different parent lines or between a variety and a wild relative. The crosses between two parental materials with different origins create more opportunities to select super lines via heterosis. Sometimes, backcross is used to transfer genetic background from the super parent to the new lines. At present, most new UGC varieties are developed via systematic selection.

#### 4.2.2. Hybrid Breeding

Hybrid breeding is popularly used in foxtail millet and sorghum in China. Generally, a hybrid of foxtail millet and sorghum can be achieved with three lines, namely the male sterile line, the maintainer line, and the restorer line. However, by using a temperature-sensitive or light-sensitive male sterile line, two-line hybrids of foxtail millet and sorghum have been successfully developed by Chinese scientists. They use male sterile lines as the female parent to cross with male parent lines that possess multi-resistance to biotic and abiotic stresses to select elite hybrids with strong heterosis [59]. 

#### 4.2.3. Mutation Breeding

Mutation breeding is the process of exposing seeds to specific chemicals or forms of light radiation, such as X-rays or gamma rays, to change the genetic composition of a plant. In recent years, ethylmethane sulfonate (EMS) mutation techniques have become well established in breeding research on foxtail millet [60] and oat [61]. Some new varieties have been developed through mutation breeding of buckwheat [62]. 

#### 4.2.4. Molecular Assistant Selection

Specific molecular markers to be used in the selection of relevant traits have been identified through constructing genetic linkage maps using different kinds of molecular markers and association analysis to explore the relationships between markers and phenotypic traits, such as the relationships between the SSR markers and grain size of oat [63] and between SNPs and the panicle traits of foxtail millet [64]. Molecular marker-assisted selection was applied in barley by linking the relevant malting QTLs/genes with high phenotypic variation in the relevant malt factors in multiple populations or environments [65] and in linking SSR markers with α-amylase activity [66].

### 4.3. National Breeding Program

The Ministry of Agriculture established the National Agricultural Research Systems in 2007. Fortunately, the six UGCs were included to form three research systems, namely (1) the Barley Research System, (2) the Buckwheat and Oat Research System, and (3) the Millets (including broomcorn/foxtail millets) and Sorghum Research System. Breeding laboratories were established for each of the three research systems by grouping key breeders from different research organizations, which is where breeding activities using the six UGCs are conducted. These research organizations are the Institute of Crop Sciences of the Chinese Academy of Agricultural Sciences (for the breeding of barley, foxtail millet, and oat), the Baicheng Academy of Agricultural Sciences (for the breeding of buckwheat and oat), the Liaoning Academy of Agricultural Sciences (for the breeding of foxtail millet and sorghum), the Heilongjiang Academy of Agricultural Sciences (for the breeding of sorghum), the Hebei Academy of Agricultural Sciences (for the breeding of foxtail millet and oat), the Shanxi Academy of Agricultural Sciences (for the breeding of buckwheat, broomcorn millet, and oat), the Yunnan Academy of Agricultural Sciences (for the breeding of barley and buckwheat), Zhejiang University (for the breeding of barley), Guizhou Normal University (for the breeding of buckwheat), and Northwest Agriculture and Forest University (for the breeding of buckwheat and broomcorn millet).

### 4.4. Release of New Varieties

The release of new varieties of UGCs in China is currently guided by the Measures for Registration of Non-Major Crop Varieties issued by the Ministry of Agriculture and Rural Affairs in 2017. During that same year, the First List of Non-Staple Crops for New Variety Registration was published. Barley, foxtail millet, and sorghum were included in the first list for variety registration. Buckwheat, broomcorn millet, and oat are still waiting for inclusion in the next list to be released. Accordingly, the new varieties that are developed by breeders should go through the process of registering with the provincial agricultural department, which is responsible for reviewing and approving registrations. The approved list of new varieties should be reported to the Ministry of Agriculture and Rural Affairs for final review before official release to the public. Since 2017, 1431 new varieties of UGCs have been registered and released by research organizations and enterprises, including 206 for barley, 573 for foxtail millet, and 654 for sorghum (Table 2). Most of the new varieties are conventional ones that can be used and kept by farmers. However, a considerable number of foxtail millet and sorghum hybrids have been developed and registered. Although no entries for new buckwheat, broomcorn millet, and oat varieties have been registered, many new varieties have been developed and made available directly to farmers.

## 5. Production

### 5.1. Plant Physiology

Seeds of UGC cereals can germinate at very low temperatures (about 4 °C), while buckwheat seeds need high temperatures (above 15 °C). Barley, buckwheat, and oat prefer cool temperatures of 20–25 °C during the growth period. It is important to understand the conditions that promote various development responses from plants and that monitor the growth and control the external inputs. Studies on the responses of barley plants to different levels of altitude have demonstrated that plant height increases, the growing period extends, and the protein content decreases as the altitude increases [67]. Studies on barley drought resistance have shown that appropriately dry soil stimulated the growth of fibrous roots that can help the plant to take more nutrients and moisture from the soil [68]. Under water stress, oat yield was significantly correlated with root soluble sugar and leaf relative electroconductibility [69]. The drought resistance indexes of foxtail millet were systematically established by the indicators of the relative root–shoot ratio, relative grain weight per ear, relative rate of photosynthesis during the filling period, and the rate of transpiration [15], while the chlorophyll, soluble sugar, malonic dialdehyde (MDA), and superoxide dismutase (SOD) contents were used as physiological and biochemical indicators [70].

### 5.2. Cultivation Technologies

During production, different cultivation techniques are adopted according to different ecological environments. Barley, foxtail millet, and sorghum have relatively high fertilizer and water inputs; as a consequence, the yield per unit is relatively high. There are lower inputs for buckwheat, broomcorn millet, and oat. In recent years, so-called light and simple approaches have been implemented in production by reducing the inputs and managing processes of production to save labor and capital, improve efficiency, and promote ecological and organic production [71]. No-tillage has also been adopted for UGCs to keep the stems and roots in the field until the next planting time, which can prevent soil from experiencing wind erosion and increase the soil organic matter. Techniques have been developed to cultivate buckwheat in high-altitude regions by selecting suitable varieties, planting at the proper time, applying organic fertilizer, and harvesting on time [72]. In recent years, good progress has been made in developing technologies for cultivating oat in saline and alkaline land, particularly in the delta region of the Yellow River [73].

### 5.3. Farming System

The diversification of planting systems, including intercropping, mixed planting, and rotation with other crops, has been adopted to improve the comprehensive benefits resulting from UGC production. Farming systems are designed according to the adaptability of the crops and the climate conditions in different areas. Generally, they are planted for one crop season in the rainfed areas in the north, northeast, and northwest of China. However, barley, buckwheat, and oat have also been also planted as the second crop in the areas of central China where rice is usually planted as the first crop. Although UGC monocultures represent the main model in most areas, intercropping using oat and potato [74], soybean/legumes [75], and sunflower [76] and intercropping between foxtail millet and peanut [77] are becoming popular in the northwest of China, and these areas effectively weaken the wind speed at the height of 20 cm near the soil surface and reduce the soil moisture evaporation from 0 to 10 cm under the soil surface, increasing the land yield by about 5% [78]. The rotation of foxtail millet with winter wheat in the Yellow River area and oat with wheat or oat with potato or beans in the northwest represents an important farming practice in China. Mixed cropping using barley or oat with forages such as alfalfa or Chinese clover is popular in Xinjiang, Qinghai, and Tibet, and this cropping system can help to enhance productivity and improve the quality of animal feed and forage.

Traditionally, UGC cultivation is mainly managed by individual households in small-scale growing situations and only involves the use of manure made of plant ash and animal waste. Sowing buckwheat by hand-throwing, a system in the southwest, particularly in Yunnan, can effectively control weeds by maintaining a high plant density on the field [79]. These kinds of cultivation systems allow for the optimal use of natural resources and sustainably maintain the fertility of the earth, benefitting the environment. The products resulting from traditional cultivation systems have higher nutritional and healthy quality. Buckwheat and oat are popularly cultivated using these ecological methods.

## 6. Nutrition and Food Processing

### 6.1. Nutritional and Functional Components

Richness in elements is an outstanding characteristic of UGCs. In addition to protein, fat, and carbohydrates, they are also rich in vitamins, dietary fiber, and minerals. UGCs have become a typical green and healthy food with a high nutritional value. In recent years, research on the nutrition of UGCs has made rapid progress, promoting the exploration and utilization of high-quality UGC germplasm resources.

#### 6.1.1. Barley

Barley grain contains about 80% carbohydrates composed of soluble sugars and insoluble sugars [80]; 11.19–17.07% protein, mainly albumin, globulin, prolamin, and gluten [81]; and 4.4% fat, of which unsaturated fatty acids account for more than 60% [82]. The content of B vitamins can reach 15.2 mg/kg, making it a high-quality source of B vitamins for humans [80]. It contains 2–3% minerals, including a macro-amount group of 0.5 g/kg Ca, 3.5 g/kg P, 4.7 g/kg K, 1.4 g/kg Mg, 0.5 g/kg Na, and 1.4 g/kg Cl and a micro-amount group of 45.7 µg/g Fe, 27.2 µg/g Mn, 34.4 µg/g Zn, and 0.4 µg/g Se [80]; many cultivars contain 4.58% β-glucan, and a variety from Tibet can have contents as high as 8.62% [83].

#### 6.1.2. Broomcorn Millet

Broomcorn millet grain contains 13.6% protein, mainly water-soluble albumin, salt-soluble globulin, and albumin [84]. The total grain amino acid content for the protein component is between 12.8% and 15.2%, while the essential amino acids are between 5.35% and 6.93%, accounting for 34.56% of the total amino acids [85]. These grains contain 35 mg/kg vitamin E, 45 mg/kg vitamin B1, and 18 mg/kg vitamin B2; the grain minerals are 1.16 mg/g Mg, 0.30 mg/g Ca, and 0.057 mg/g Fe [84].

#### 6.1.3. Buckwheat

Tartary buckwheat grain contains 8.5–12.5% protein, 2.39–3.45% fat, and 67.59–77.59% starch; the cellulose content varies from 6.64% to 10.2% between varieties from different regions, with insoluble dietary fiber accounting for about 80% of the total cellulose content [86]. The contents of trace elements are 7.19 mg/kg Cu, 13 mg/kg Zn, 32 mg/kg Fe, 18.7 mg/kg Mn, 0.746 mg/kg Cr, 10.2 mg/kg Ni, and 20.5 mg/kg Se [87]. Flavonoids are important bioactive substances in Tartary buckwheat. Studies have shown that the total flavonoid content is 1.13–1.63% [86] and as high as 2.40% in some varieties [88]. 

#### 6.1.4. Foxtail Millet

The average protein content of foxtail millet is 11.42%, ranging from 9.73% to 14.93%; the essential amino acid content is reasonable and higher than that of staple crops, with the exception of the lysine content; the average fat content is 4.28%, ranging from 3.20% to 6.41%, among which the unsaturated fatty acids accounts for 85%, ranging from 78% to 90% [84,89,90]. The carbohydrate content in the grains is 72.8%, while the contents of vitamin A and vitamin B1 are 1.9 mg/kg and 6.3 mg/kg, respectively [84]. These grains contain 20.8 ug/kg–89.2 ug/kg Se [91], 2.67 mg/g–3.08 mg/g K, 0.78 mg/g–1.09 mg/g Mg, 0.79 mg/g–0.92 mg/g Ca, and 0.16 mg/g–0.30 mg/g Zn [92].

#### 6.1.5. Oat

Oat germplasm grain contains 11.9–20.50% protein and 3.44–9.82% fat [93], while oat cultivars contain 10.68–16.65% protein and 5.15–7.54% fat [94]. Generally, oat grain contains 0.15 g/kg vitamin E, 5.90 mg/kg vitamin B1, 1.50 mg/kg vitamin B2, and 1.60 mg/kg vitamin B6; the mineral contents in oat grains are 0.55 g/kg Ca, 3.35 g/kg K, 0.05 g/kg Fe, 0.05 g/kg Mn, and 0.04 g/kg Zn [84]. Oat germplasm contains more β-glucan, ranging from 2.5% to 7.5% [93], while oat cultivars have 2.74–5.72% [95].

#### 6.1.6. Sorghum

Sorghum contains 74.7% carbohydrates, with amylose accounting for 70–80% and amylopectin accounting for 20–30%; it contains 10.4% protein, with the protein content ranging from 6% to 18% [96]. There is a wide range of amino acids in sorghum grains, including essential amino acids for the human body. The grain fat content is 3.0%, ranging from 2.5% to 3.8% [97]. It is rich in vitamins B1 and B6. The contents of calcium, iron, zinc, magnesium, and selenium are 22 mg/kg, 63 mg/kg, 16.4 mg/kg, 1290 mg/kg, and 28.3 mg/kg, respectively [96].

### 6.2. Traditional Foods and Processed Products

#### 6.2.1. Traditional Foods

UGCs can be used to prepare many traditional or local foods [8]. Examples include bowl jelly and jelly noodles, which are made from buckwheat in Shanxi; kaolou, which is made from buckwheat flour and the sour rice from broomcorn millet in Shaanxi; hele and boyu noodles, which are from buckwheat; steamed rolls, which are made from oat; fried rice made from broomcorn millet in Inner Mongolia; zanba, which is made from barley in Tibet; and battercake, which is from foxtail millet in Shandong. These traditional foods made using UGCs are still very popular in the local markets and play an important role in enriching the food diversity in China.

#### 6.2.2. Primary Products

Raw UGC grains are simply processed for primary products, such as husked grains and flour [98]. Husked grains of barley, broomcorn millet, buckwheat, foxtail millet, oat, and sorghum are prepared for use as foods through steaming or boiling in water. Buckwheat flour is made into fresh and dried noodles and steamed bread, which are popular recipes that are commonly prepared by Chinese people. Mixed grains from buckwheat and millet are used to cook porridge and as mixed flour to make battercake [99]. Most UGC grains that are harvested in China go through a primary process and are made available at local markets and supermarkets in cities.

#### 6.2.3. Instant Foods

UGC grains are processed into bread, biscuits, and instant noodles, with examples including bread, cake, peach crisp, and biscuit made from buckwheat; fried powder, biscuits, and oatmeal made from oat; cake, wafer, crisp rolls, and biscuits from foxtail millet; and fried rice made from broomcorn millet [96,98]. Instant foods prepared using UGCs are popular, and there is an increased market demand for these products.

#### 6.2.4. Brewed Drinks

UGC grains can be brewed to make two kinds of drinks: ordinary beverages and fermented beverages. Ordinary beverages include buckwheat tea, barley tea, barley coffee, oat milk, and millet milk; fermented beverages include Tartary buckwheat wine and vinegar, barley wine and beer, and millet wine and sorghum wine [99,100]. Generally, grains from different UGCs are brewed together to make wine, something that is enjoyed by the Chinese population. 

### 6.3. Marketing Potential

#### 6.3.1. Consumption Habit Changing

In recent years, the consumption ability and habits of the Chinese population have experienced continued change, from eating more refined food to going back to more UGC- and vegetable-based foods. Previously, it was determined that dietary habits favoring refined food and less miscellaneous grains pose a serious threat to people’s health. The low intake of vegetarian protein and UGCs has resulted in the prevalence of constipation, obesity, hypertension, hyperlipemia, and hyperglycemia. It has been reported that 30.6% of Chinese people over 18 years old are overweight and that the prevalence of hypertension was 25.2% and the prevalence of diabetes was 9.7% [101]. The seriousness of the above-mentioned problems has been recognized, and people’s dietary habits are changing, and they are consuming more UGCs in their diets.

#### 6.3.2. Demand Increasing

With the increase in UGC consumption, market demand will continue to increase. To meet the increased demands of local markets, considerable amounts of UGCs have been imported, with an increasing trend being observed from 2019 to 2021 (Table 3). Barley is the largest import, reaching over 12 million tons in 2021, followed by sorghum and oat. Only millets are not imported. In contrast, only small amounts of UGCs are exported. More categories and quantities of various processed UGC products are made available in markets. As processing technology improves, the taste of UGC products has been greatly improved. There are not only primary processed products such as grain rice and noodles but also deeply processed products such as bread, biscuits, and even functional foods suitable for the elderly and children, and these products have caused the domestic market demand to grow rapidly. It is expected that the annual growth rate of the market demand for UGC products will exceed 10%.

#### 6.3.3. Active Trade

Local farmers have started to use UGCs as a source of income by selling the grains that are produced. The trade of UGC commodities has been increasing in recent years. Before the end of the 20th century, the UGC trade was basically carried out by individual merchants based on the local market and gradually moved toward urban and rural markets. At present, many UGC wholesalers, logistics distributors, and various exhibition fairs have participated in UGC trading. The market is very active, and trade volume has increased year by year. UGC retail is also developing rapidly. In addition to urban supermarkets and food and oil shops, many e-commerce companies have established online sales platforms for UGC products, making it convenient for consumers to buy UGC products. More and more UGC foods are being put on the tables of families and restaurants and are even being chosen by people as gifts.

### 6.4. Benefits

#### For Producers

Growing demands from consumers in urban areas for diverse and novel foods are creating new market niches for underutilized species. These market opportunities generate more income for farmers, who largely depend on these crops for their livelihoods. Generally, UGCs have great price advantages over staple crops. Although the yield of UGCs per unit area is only 30–50% that of staple crops, their marketing price is 2–3 times that of staple crops. As no chemical fertilizers and pesticides are used, the productive costs of UGCs are significantly lower. Through the industrialization cooperation mode of “companies + cooperatives + farmers”, the growing areas of UGCs have been extended and product quality has been advanced in key producing regions. UGCs have become important options for generating more income and improving the livelihoods of local farmers [102].

### 6.5. For Consumers

UGCs are critical sources of various macro- and micronutrients, which ensure nutrient adequacy in human food. Each of the six UGCs has its own characteristics and can be made into a variety of foods, including processed foods and traditional foods. They not only provide various nutrients needed by the human body but also help to maintain the nutritional balance of the human body and improve malnutrition by preventing obesity and overweight. A study showed that the intake of food made from the whole grains of UGCs improves nutrient levels through the increased intake of dietary fiber, carbohydrates, vitamin B1, niacin, magnesium, and potassium He et al. 2016. Crops with a content of dietary fiber such as barley, buckwheat, and oat play a function of improving health not only by helping in weight loss [103] but also by reducing cholesterol and preventing hypertension and hyperlipidemia [104].

### 6.6. For Environments

Chemical fertilizer and pesticides have become critical factors causing agricultural non-point-source pollution [105]. The application of chemical fertilizers and pesticides on UGCs is far lower than that on staple crops. Generally, UGCs make more effective use of natural resources and are managed in a more environmentally friendly way. The production of buckwheat and millet is mainly based on farm manure. The amount of chemical fertilizer applied to the other five UGCs is only 30–50% of that applied to staple crops. UGCs are also an important part of the agricultural ecosystem in the mountainous areas of the north, northwest, and southwest of China and play a very important role in preventing soil erosion and promoting the resiliency of the agro-ecosystem in these regions. As the whole process from the field to the table is short, the production and marketing of UGCs consume less energy and cause low carbon emissions, which is conducive to environmental protection. It is indicated that oat performs well in soil with a pH value between 5.5 and 6.5 and that it helped to increase the vegetation coverage rate of saline–alkali land in China [106].

## 7. Conclusions

China is rich in UGCs and has plentiful germplasm resources. UGCs have played very important roles in food security in Chinese history. In the past half-century, although the research and development efforts on UGCs have often been overshadowed by those on staple crops such as rice, wheat, and corn, progress in research and development has been made in the germplasm conservation, breeding, cultivation, and processing of UGCs. Over 85,000 accessions have been conserved for future utilization. Genetic diversity has been identified to understand the origin, evolution, and relationships between the genetic materials in UGCs. In the last five years, over 1400 new advanced varieties have been developed and released to farmers through the national variety registration system for underutilized crops. UGC production has been promoted by understanding plant physiological responses to climates, soil, and biotic and abiotic presses; developing cultivation technologies by optimizing supplies of water and fertilizers; and adopting the intercropping and rotation farming systems. Traditional and local foods as well as nutritional and healthy products made using UGCs meet the diversified demands found in domestic and international markets. The adequate use of UGCs represents a benefit for producers, consumers, and environments. However, the UGC research and development still face the challenges of inadequate support from national policies and investments. Recognizing the roles of UGCs in promoting nutrition and green agriculture, and responding to climate change, efforts are being made to strengthen the conservation and sustainable use of germplasm resources, including the protection of wild relatives in situ and local varieties on-farm, and to improve the productivity of the new varieties and nutritional components in processed products. Value chains could represent an effective way to involve relevant stakeholders and to link farmers to markets to generate more income and contribute to food and nutritional security in China.

## Figures and Tables

**Table 1:** UGC accessions stored in the National Crop Genebank [21].

Crop Names	Accessions (1000)
Barley	22.6
Buckwheat	3.3
Broomcorn millet	6.0
Foxtail millet	28.4
Oat	4.8
Sorghum	20.7
Total	85.8

**Table 2:** Varieties of UGCs registered and released since 2017.

Crop	2017	2018	2019	2020	2021	Total
Barley	10	103	22	57	14	206
Foxtail millet	27	260	115	132	39	573
Sorghum	36	263	112	186	55	652
Total	73	626	429	375	108	1431

**Table 3:** Data on import and export of six UGCs during 2019–2021 (tons) *.

	2019		2020		2021	
	Import	Export	Import	Export	Import	Export
Barley	5,928,689	315	8,079,493	78	12,480,100	89
Buckwheat	36,506	22,624	3388	13,169	27,529	7977
Broomcorn/foxtail millets	0	6145	0	5302	0	4955
Oat	239,408	134	233,083	171	335,391	152
Sorghum	831,943	41,048	4,813,139	21,209	9,416,401	3637
Total	7,036,546	70,267	13,129,103	39,930	22,259,421	16,810

* Data were obtained from the website of the General Administration of Customs of the People’s Republic of China (http://www.customs.gov.cn/, accessed on 29 August 2022).
